# Dark halo, a new biomarker in macular neovascularization: comparison between OCT angiography and ICGA—a pilot prospective study

**DOI:** 10.1007/s00417-022-05693-8

**Published:** 2022-05-06

**Authors:** Federica Fossataro, Gilda Cennamo, Daniela Montorio, Lidia Clemente, Ciro Costagliola

**Affiliations:** 1 Department of Neurosciences, Reproductive Sciences and Dentistry, University of Naples “Federico II”, Naples, Italy; 2 Eye Clinic, Public Health Department, University of Naples “Federico II”, Via S. Pansini 5, 80133 Naples, Italy

**Keywords:** AMD, Choriocapillaris flow deficit, Dark halo, ICGA, Macular neovascularization, OCTA

## Abstract

**Purpose:**

To compare optical coherence tomography angiography (OCTA) and indocyanine green angiography (ICGA) in terms of reliability in detecting dark halo in patients affected by age-related macular degeneration (AMD) complicated with type 1 macular neovascularization (MNV).

**Methods:**

Eighty-nine eyes of 89 patients were analyzed at the University of Naples Federico II between January 2018 and October 2021. Each patient underwent a complete ophthalmological evaluation including fluorescein angiography, ICGA, spectral domain optical coherence tomography (SD-OCT), and OCTA. OCTA and ICGA images of dark halo were compared. The paired Student’s test and intraclass correlation coefficients were used to evaluate the differences in dark halo measurements between OCTA and ICGA images.

**Results:**

Thirty-six eyes of 36 patients were included in this prospective study. Dark halo area was significantly larger in OCTA than in ICGA (1.49 ± 1.8 mm^2^ vs. 0.54 ± 0.5 mm^2^; *p* = 0.001). Moreover, the agreement between the two types of devices for measuring dark halo areas was poor, with a low intraclass coefficient correlation (0.397).

**Conclusion:**

OCTA could be a useful and necessary tool to investigate dark halo in neovascular AMD due to its ability to visualize the areas of reduced vessel density around MNV in greater detail compared to ICGA.

**Clinical trial registration:**

ClinicalTrials.gov Identifier: NCT05108285

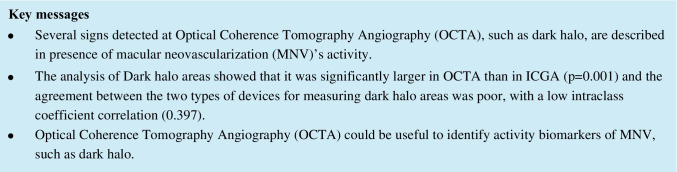



## Introduction

Age-related macular degeneration (AMD) is a progressive retinal disease that represents the most common cause of legal blindness in developed countries in patients over 55 years of age [1]. AMD complicated with macular neovascularization (MNV) is one of the two advanced forms of AMD, along with geographic atrophy [2, 3].

Until a few years ago, MNV identification was based on the use of both fluorescein (FA) and indocyanine green angiography (ICGA) [4, 5]. ICGA is a consolidated technique used to study choroidal circulation, due to the high binding (98%) of the dye to plasma proteins, and consequently to limit diffusion through choriocapillaris [6].

With the introduction of optical coherence tomography angiography (OCTA), an innovative and non-invasive technique, blood flow in the retina and choriocapillaris can now be analyzed with far greater depth than before [7]. Additionally, OCTA plays a meaningful role in standardizing the current nomenclature for reporting MNV [2].

Although AMD pathogenesis is not yet fully understood, several studies have described the development of MNV, associated with microvasculature choriocapillaris flow deficits in the area surrounding the neovascular lesion [8–10]. Rispoli et al. [11] quantified choriocapillaris vascular density changes, renamed *dark halo*, around MNV before and after anti-vascular endothelial growth factor (VEGF) injections using OCTA. In particular, the authors showed a dark halo fluctuation after intravitreal injections, hypothesizing its role as a biomarker of MNV activity [11].

Dark halo can be detected by either ICGA or OCTA [12, 13]. The former reveals a distinctly dark edge surrounding the MNV until the late stages of the angiography. OCTA detects choriocapillaris flow deficits in the perilesional area, which appear more evident in active MNV [11, 14, 15].

This prospective study aimed to quantify dark halo in patients with AMD complicated with type 1 MNV using ICGA and OCTA and to compare both techniques in terms of reliability in detecting this new activity biomarker.

## Materials and methods

During the enrollment period (from January 2018 to October 2021), 89 eyes of 89 subjects affected by exudative AMD were recruited to the Eye Clinic of the University of Naples Federico II.

The criteria for inclusion were age greater than 50 years and diagnosis of treatment-naïve exudative AMD due to the presence of type 1 MNV.

The exclusion criteria were MNV secondary to causes other than AMD, idiopathic polypoidal choroidal vasculopathy (PCV), retinal angiomatous proliferation (RAP), type 2 MNV, previous intravitreal injections of anti-VEGF for CNV, geographic atrophy, subretinal fibrosis, vitreoretinal diseases, retinal vascular diseases, myopia greater than 6 diopters, history of intraocular surgery, and significant lens opacity. We also excluded images with visible eye motion or blinking artifacts and low-quality images obtained with OCTA.

Each patient underwent a complete ophthalmological evaluation, including the evaluation of best-corrected visual acuity (BCVA) according to the Early Treatment of Diabetic Retinopathy Study (ETDRS), slit-lamp biomicroscopy, applanation tonometry, fundus examination, FA, ICGA (Spectralis, Heidelberg Engineering, Heidelberg, Germany), spectral domain (SD)-OCT (Spectralis, Heidelberg Engineering, Heidelberg, Germany), and OCTA (AngioVue, RTVue XR Avanti, Optovue, Inc., Freemont, CA).

The study was registered on ClinicalTrials.gov (NCT05108285), and all investigations adhered to the tenets of the Declaration of Helsinki. Written informed consent was obtained from the patients enrolled in the study.

### Optical coherence tomography angiography

OCTA images with the Optovue Angiovue System (software ReVue XR version 2017.1.0.151, Optovue Inc., Fremont, CA, USA) were performed following a standardized protocol based on the split spectrum amplitude decorrelation algorithm (SSADA), as previously described [16].

The AngioAnalytic^TM^ software automatically calculated the vessel density (VD) of the choriocapillaris on a 6 mm × 6 mm macular area. The VD was defined as the percentage area occupied by the microvasculature in the whole scan area and in all sections [17].

The 3D Projection Artifact Removal (PAR) algorithm was performed to improve the quality of OCTA images. For each eye analyzed, the software automatically elaborated the vessel density in the whole scanned area and in all sections of the grid in the choriocapillaris region selected by the operator in retinal angiogram (between upper: Bruch membrane offset − 9 μm and lower: Bruch membrane offset 31 μm).

Excluded from the analysis were images with a signal strength index of less than 80, residual motion artifacts, incorrect segmentation, or low centration or focus.

### Dark halo assessment

Dark halo is defined as a choriocapillaris area, surrounding the MNV, characterized by a flow deficit detected by OCTA. The hypofluorescent halo (dark edge of hypofluorescence) around the MNV is also present in the early phase of ICGA and could correspond to the area of dark halo identified by OCTA.

To measure dark halo areas, OCTA and ICGA images were assessed separately, independently by two ophthalmologists (FF and LC). In case of disagreement, a third senior retinal specialist (GC) was asked to evaluate the image.

Firstly, the authors collected and analyzed both choriocapillaris flow density images and scan segmentation by OCTA. The blue space area around the MNV corresponding to dark halo was measured using ImageJ software (Version 1.50i; National Institutes of Health, Bethesda, MD, USA) as previously described [11]. Before the analysis, we used “the set scale” selection tool, entering the known distance and the unit of measurement in pixels to scale the image in millimeter square. The ophthalmologists converted any OCTA images to 8-bit images and then selected the *adjust threshold* function with the intensity threshold set from 0 to 50 for choriocapillaris OCTA images. The red pixel area around the MNV corresponded to dark halo, and it was manually identified and automatically quantified with the “measure tool” by ImageJ software and collected for statistical analysis.

The readers also collected ICGA images, used for delineation of the dark halo area, taken within 1–4 min after dye injection, according to a previous study [18]. Similar to the analysis performed with OCTA, the ICGA image was manually detected and automatically computed in ImageJ.

**Fig. 1:**
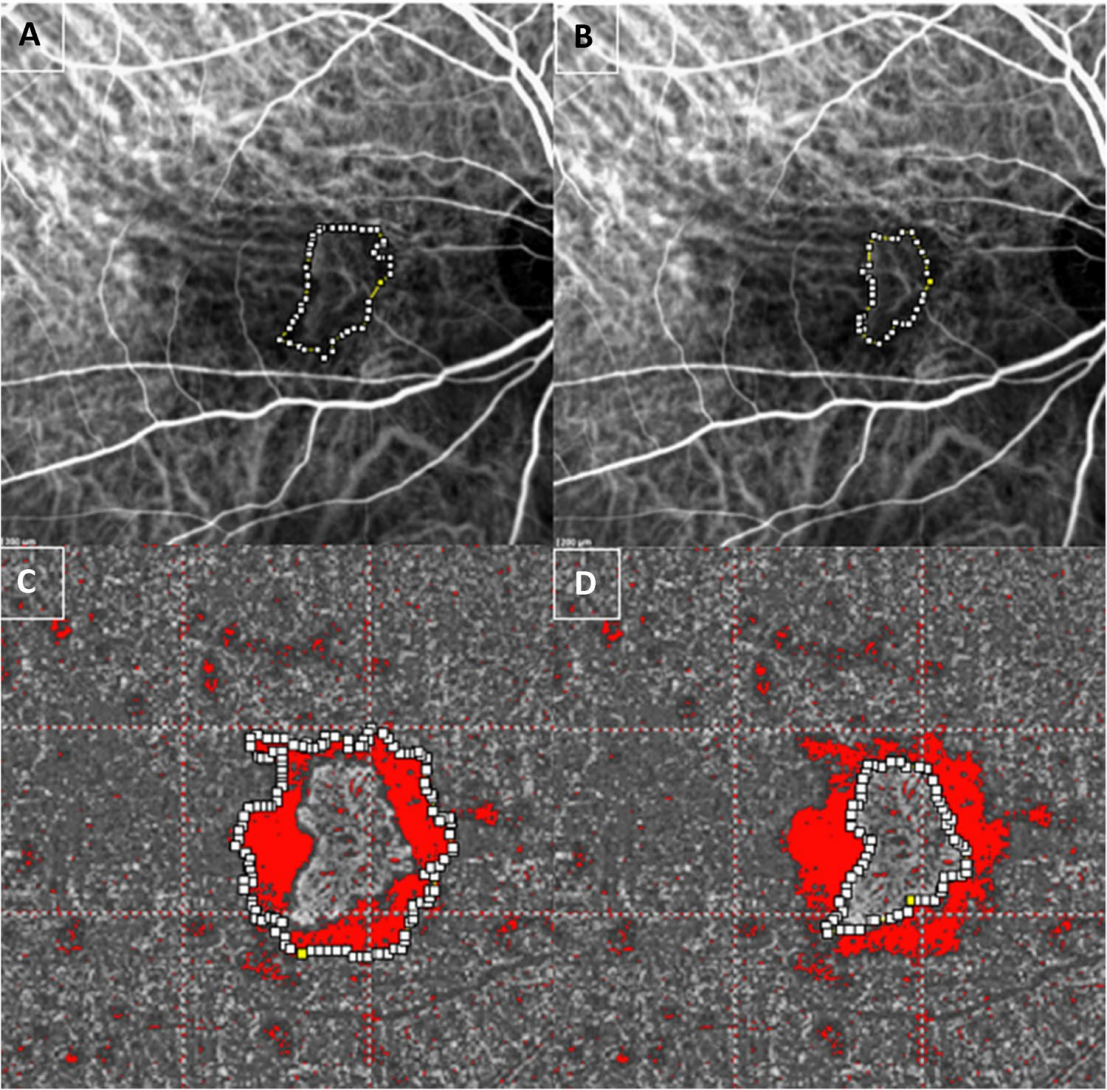
Top row. Right eye of a patient with age-related macular degeneration (AMD) complicated with type 1 macular neovascularization (MNV). The whole area (including MNV and dark halo area) and the MNV at indocyanine green angiography (ICGA) were manually selected and automatically measured using ImageJ (**A**, **B**). The whole area and the MNV at optical coherence tomography angiography (OCTA) were manually detected and automatically measured using ImageJ (**C**, **D**). The difference between the whole area and the MNV corresponds to the dark halo

For both OCTA and ICGA images, the whole area, including the MNV and surrounding dark halo, was detected manually and measured automatically using ImageJ. Then, the contour of the MNV area was delineated point by point and measured in millimeter square. On binarized images, the dark halo was calculated as the difference between the whole area and the MNV area (Fig. 1).

### Statistical analysis

Statistical analysis was performed with SPSS (Version 25 for Windows; SPSS Inc, Chicago, IL, USA). The Shapiro–Wilk test confirmed that all variables were normally distributed. Continuous variables are expressed as mean ± standard deviation (SD). The paired Student’s test was used to evaluate the differences in the dark halo measurements between OCTA and ICGA images. The intraclass correlation coefficients (ICCs) and 95% CIs were used to assess the absolute agreement between the dark halo area measurements from different types of OCTA and ICGA scans. ICC values of < 0.5, between 0.5 and 0.75, between 0.75 and 0.90, and > 0.90 indicated poor, moderate, good, and excellent agreement, respectively. A *p*-value of < 0.05 was considered statistically significant.

## Results

From January 2018 to October 2021, 53 out of 89 eyes were excluded because of poor traditional angiography and OCTA images due to low signal strength. A total of 36 eyes of 36 patients were included in this prospective study (mean age 75 SD ± 10 years; 14 women and 22 men).

From choriocapillary slab OCTA, the mean whole area (MNV + dark halo) was 2.01 SD ± 2.4 mm^2^, and the MNV area was 0.57 SD ± 1 mm^2^.

From ICGA scans, the mean whole area (MNV + dark halo) was 1.81 SD ± 2.5 mm^2^, and the MNV area was 1.27 SD ± 2.1 mm^2^.

**Fig. 2:**
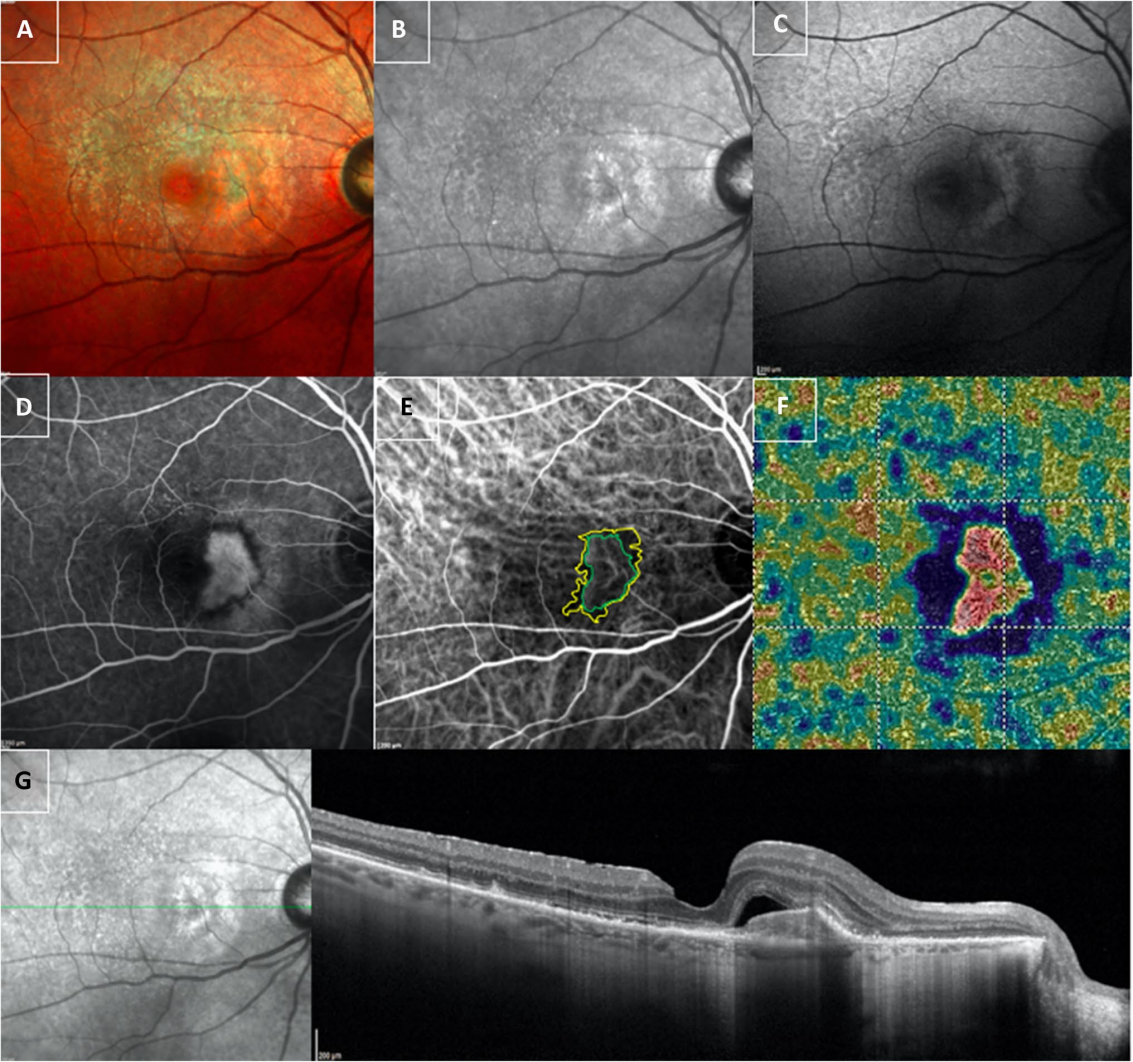
Top row. Right eye of a patient with age-related macular degeneration (AMD) complicated with type 1 macular neovascularization (MNV). Multicolor (**A**) and infra-red (**B**) examination showed increased macular thickness associated with drusen and reticular drusen. Autofluorescence image (**C**) detected a hyper-autofluorescent area in the macular region. Middle row. Fluorescein angiography showed a hyperfluorescent area at the posterior pole (**D**). Indocyanine green angiography detected a hyperfluorescent vascular network (green line) with a dark halo surrounding (yellow line) (**E**). Choriocapillaris vessel density map, at the optical coherence tomography angiography (OCTA) scan, showed a wide dark halo around the macular neovascularization (**F**). Bottom row. Spectral domain optical coherence tomography (SD-OCT) detected drusen, pseudodrusen, and an increased macular thickness associated with subretinal fluid and pigment epithelium detachment in the macular region (**G**)

**Fig. 3:**
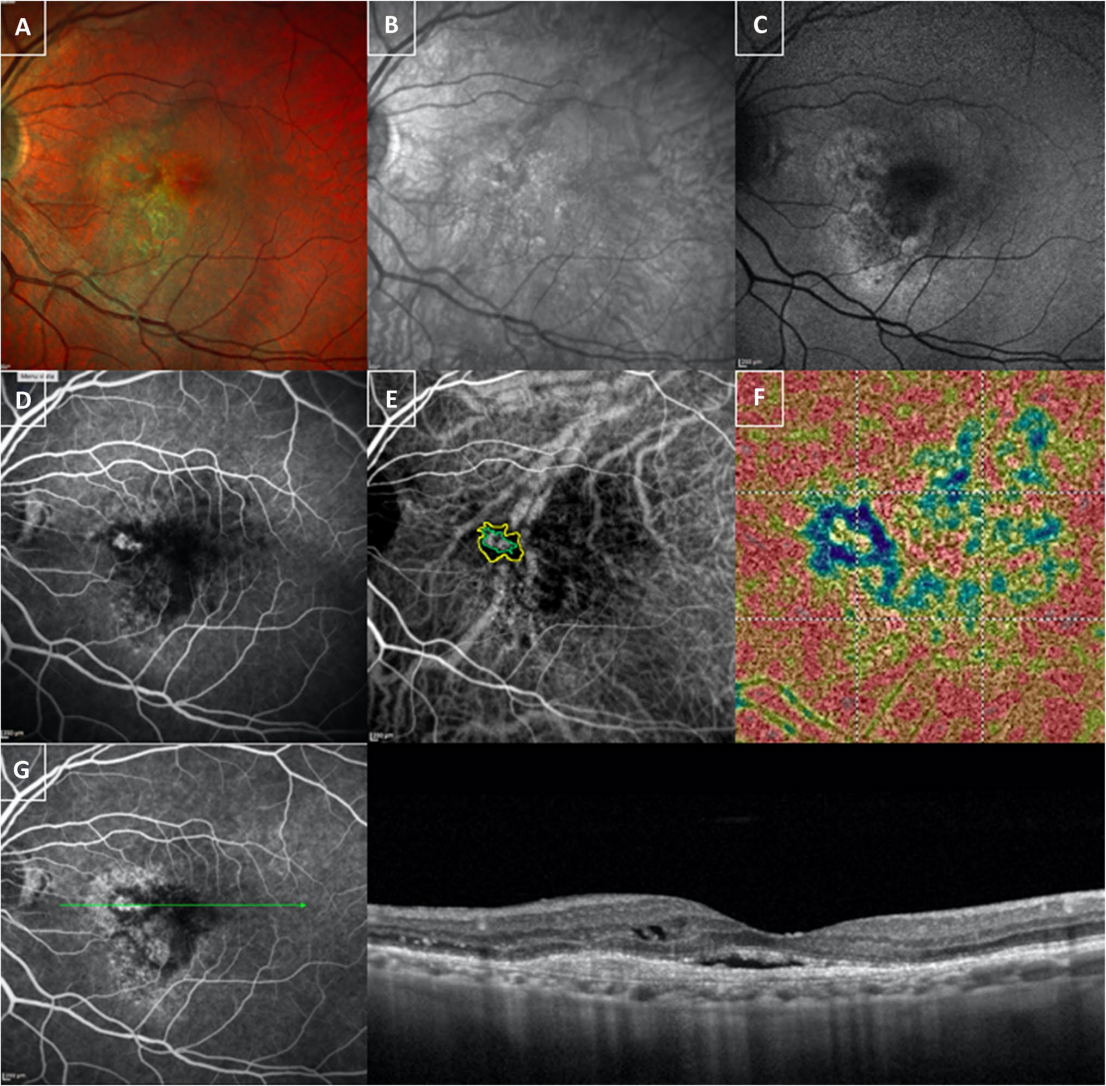
Top row. Left eye of a patient with age-related macular degeneration (AMD) complicated with type 1 macular neovascularization (MNV). Multicolor (**A**) and infra-red (**B**) examination showed macular hemorrhage with retinal pigment epithelium irregularities. Autofluorescence image (**C**) detected a hypo/hyper-autofluorescent area in the macular region. Middle row. Fluorescein angiography showed a stippled hyperfluorescence at the posterior pole (**D**). Indocyanine green angiography detected a hyperfluorescent vascular network (green line) with a dark halo surrounding (yellow line) (**E**). Vessel density of the choriocapillaris showed dark halo around the macular neovascularization at the optical coherence tomography angiography (OCTA) (**F**). Bottom row. Spectral domain optical coherence tomography (SD-OCT) detected a wide hyperreflective pigment epithelium detachment, with subretinal and intraretinal fluid (**G**)

The dark halo areas on OCTA images were statistically significantly larger than those measured at ICGA scans (1.49 SD ± 1.8 mm^2^ vs. 0.54 SD ± 0.5 mm^2^; *p* = 0.001; Table 1; Figs. 2 and 3).

**Table 1:** Demographic and clinical characteristics of patients affected by macular neovascularization

Eyes (*n*)	36
Female/male	14/22
Age (years)	75 ± 10
MNV OCTA (mm^2^)	0.57 ± 1
MNV ICGA (mm^2^)	1.27 ± 2.1
Dark halo OCTA (mm^2^)	1.49 ± 1.8
Dark halo ICGA (mm^2^)	0.54 ± 0.5

Data are expressed as mean ± standard deviation; *MVN*, macular neovascularization; *OCTA*, optical coherence tomography angiography; *ICGA*, indocyanine green angiography

The agreement between the two types of scans for measuring the dark halo areas was poor, with an intraclass correlation coefficient of 0.397 (95% CI: − 0.090 to 0.679).

## Discussion

The early identification of activity signs of MNV before they are evident on structural OCT remains a challenge. Nowadays, OCTA plays a crucial role in the diagnosis and management of neovascular AMD due to its reliability in detecting and monitoring retinal and choriocapillaris microvascular changes in affected patients. The proper method of studying type 1 MNV consists of analyzing the neovascular network and its activity signs (including dark halo) at the choriocapillaris segmentation slab. In this study, we first ruled out any artifacts for the two diagnostic techniques to properly quantify the dark halo on OCTA and ICGA as previously described [19].

Although some artifacts in the assessment of dark halo by OCTA have been reported, several studies have observed that its presence is attributable to lower choriocapillaris flow [15, 20]. Jia et al. [15] hypothesized that the area of choriocapillaris ischemia, associated with AMD, may influence the pathogenesis of MNV, which could develop to compensate for the reduced circulation. Moult et al. [20] and Coscas et al. [21] also observed the presence of a halo of choriocapillaris flow deficit located around the MNV on OCTA. These findings were also confirmed by the histologic studies of McLeod et al. [22] and Lutty et al.[23]. The authors described the loss of choriocapillaris around MNV, associated with Bruch’s membrane deposits. They also asserted that changes in the retinal pigment epithelium (RPE), involved in the growth and regression of MNV, could be associated with loss in the choriocapillaris. However, the choriocapillaris flow deficits have been described even in areas with intact RPE, suggesting that loss in choriocapillaris may be the first insult in neovascular AMD [14].

Moreover, according to Seddon et al. [24] who described that histopathologic choriocapillaris changes might influence the production of VEGF causing the growth of the MNV, Rispoli et al. [11] reported dark halo fluctuation after intravitreal injections. The authors suggested that the development of the MNV could induce a decreased flow in the surrounding choriocapillaris due to blood deviation and blood sequestering. Furthermore, they observed that dark halo reached its minimal surface between 6 to 13 days after anti-VEGF injection. In this prospective study, we enrolled naïve exudative AMD patients to detect and quantify the OCTA and ICGA dark halo before the anti-VEGF treatments.

On ICGA, until the late phase of the exam, a sharp dark rim around the MNV was described in a previous study [13]. However, until now, dark halo was observed and described in detail only with OCTA.

Therefore, the goal of our study was to compare the dark halo measurements on OCTA with the ones obtained on ICGA in naïve type 1 MNV in AMD patients in terms of reliability in detecting this new activity biomarker.

The dark halo areas were measured in all eyes with both techniques. Our results showed that the dark halo area was larger on OCTA than ICGA, with poor agreement reflected by the intraclass coefficient correlation. Many causes could explain this finding. First, ICGA analysis of choroidal circulation is performed on the full choroid since the ICGA is a two-dimensional examination, without the possibility of abstracting only the image of the choriocapillaris slab. However, with OCTA, proper segmentation is possible, aiming to study the single plexus and predefined slab that could therefore better highlight the whole area (MNV + dark halo).

Furthermore, OCTA’s greater resolving power and ability to visualize in greater detail the neovascular membrane and areas of reduced VD around the MNV could better clarify our results.

The OCTA larger dark halo area further strengthens the role of OCTA in identifying clinical activity signs of MNV and supports the hypothesis of blood sequestration present during the development of the neovascular network at the expense of the surrounding choriocapillaris areas.

This study suffers from some limitations that should be acknowledged. First of all, we enrolled small number of patients; thus, studies with a larger sample size are needed to confirm these primary results. Moreover, possible error in manually marking the area could not have been avoided.

In the future, the comparison between dark halo changes after anti-VEGF on OCTA and ICGA could be useful to better understand the pathogenesis of neovascular AMD and confirm its key role as an activity biomarker.
